# Facilitators and barriers of 2-1-1 HIV pre-exposure prophylaxis

**DOI:** 10.1371/journal.pone.0251917

**Published:** 2021-05-20

**Authors:** Christina Camp, Parya Saberi

**Affiliations:** Division of Prevention Science, University of California San Francisco, San Francisco, California, United States of America; Washington University in Saint Louis, UNITED STATES

## Abstract

An alternative strategy for men who have sex with men (MSM) experiencing challenges with daily HIV pre-exposure prophylaxis (PrEP) includes 2-1-1 dosing. Understanding 2-1-1 PrEP facilitators and barriers, especially during the SARS-CoV-2 pandemic, may guide researchers and healthcare providers in future studies and clinical preparedness. We conducted a national cross-sectional study of MSM in the US who had taken 2-1-1 PrEP to examine facilitators and barriers of this on-demand PrEP dosing option. With the shelter-in-place orders in March 2020, this study was adapted to include questions on how the SARS-CoV-2 pandemic affected participants’ PrEP use. A total of 140 individuals participated in the survey, 106 of which completed questions pertaining to the SARS-CoV-2 pandemic. The most common reasons for switching from once-daily to 2-1-1 PrEP included having sex less frequently (63.6%) and wanting to take fewer pills (46.4%). Participants reported high medication adherence based on each component of 2-1-1 PrEP dosing (>84%). The most common barriers with 2-1-1 PrEP dosing included unplanned sexual encounters resulting in missing the double-dose pre-sex (43.6%) and trouble remembering doses post-sex (29.3%). Facilitators of the 2-1-1 PrEP dosing strategy included reductions in sexual encounters (63.6%), preference to take fewer pills (46.4%), need to reduce cost (22.1%), and desire to reduce side effects (19.3%). Challenges to receiving PrEP services during the pandemic included obtaining laboratory testing (25.5%) and PrEP refills (either receipt of a refill authorization from a healthcare provider or processing of a refill from the pharmacy) (18.9%). 2-1-1 PrEP is an effective HIV prevention method; therefore, understanding facilitators and barriers of this dosing strategy can result in continuous provision of HIV prevention efforts, particularly during a pandemic.

## Introduction

In 2012, the US Food and Drug Administration approved a fixed-dose tablet of tenofovir disoproxil fumarate and emtricitabine (TDF/FTC) to prevent HIV [1]. Numerous trials have indicated that daily oral HIV pre-exposure prophylaxis (PrEP) is safe, well-tolerated, and effective in preventing HIV [2–4]. PrEP efficacy is positively correlated with PrEP adherence [5]. However, due to limited uptake, retention in care, and “pill fatigue,” novel PrEP dosing strategies for those with differing sexual health needs have been introduced [6].

2-1-1 PrEP (also known as “on-demand”, “episodic,” “event-based” or “event-driven” PrEP) entails two TDF/FTC tablets taken between 2–24 hours before sex and two additional single TDF/FTC doses taken 24 and 48 hours after the pre-sex double-dose [7]. In 2015, the IPERGAY trial showed that 2-1-1 PrEP prevented HIV transmission in 400 men who have sex with men (MSM). IPERGAY purports that episodic PrEP could “improve adherence, safety, cost effectiveness, and make PrEP more attractive [8].” Additionally, in a cohort study, it was determined that 2-1-1 PrEP was highly effective at preventing HIV among MSM; therefore, representing an alternative to daily PrEP [7].

In a retrospective study, early adopters of 2-1-1 reported on-demand dosing as an appealing alternative to daily PrEP largely due to a reduction in sex [9, 10], and less frequent missed doses and side effects compared to daily PrEP [9]. Despite the evidence of the effectiveness of 2-1-1 PrEP [8], the Centers for Disease Control and Prevention (CDC) has not endorsed this dosing strategy [11]. However, the World Health Organization (WHO) offers 2-1-1 as an alternative dosing strategy and the option of switching from daily to 2-1-1 dosing [12].

2-1-1 PrEP has found to be effective at preventing HIV acquisition for MSM, who in the IPERGAY trial reported a median of 10 sex acts per month and took a median of 15 pills per month [8]. 2-1-1 PrEP could potentially improve the PrEP care continuum for these individuals; however, there are barriers with this dosing strategy. Namely, 2-1-1 PrEP studies have mostly included MSM and a small number of TGW. Data regarding the efficacy of 2-1-1 PrEP for transgender persons, heterosexual couples, women, and intravenous drugs users is limited [13–17].

The 2018 International Antiretroviral Society-USA (IAS-USA) guidelines indicate 2-1-1 PrEP as an alternative to daily PrEP for MSM with infrequent sexual exposures which is not recommended for patients with active hepatitis B infection because of the risk of hepatic flare [18]. For those who can take 2-1-1 PrEP, it is imperative to be able to anticipate and/or negotiate the time of sex with at least a moderate level of certainty, adhere to the pre- and post-sex doses, and use this strategy at each sexual encounter [8, 19]. Therefore, understanding facilitators and barriers to 2-1-1 PrEP dosing, preferences for PrEP regimens, and the impact of shelter-in-place orders on 2-1-1 PrEP use during the Severe Acute Respiratory Syndrome Coronavirus 2 (SARS-CoV-2) pandemic may guide researchers in the considerations for future PrEP studies and assist healthcare providers in clinical preparedness and patient education.

## Methods

We conducted a national cross-sectional study of MSM in the US who had taken 2-1-1 PrEP to examine facilitators and barriers of this dosing strategy. All research activities were conducted remotely via text, telephone, and email, to allow for national recruitment [7]. Participants were recruited via flyers and in-person at a San Francisco-based sexual health clinic, through correspondence with clinicians and clinic staff, and by posting on PrEP listservs, social media outlets, and dating apps including Facebook, Scruff, and Grindr. We received the University of California, San Francisco (UCSF) Institutional Review Board approval to conduct this study using verbal informed consent.

HIV-negative MSM, ≥18 years of age, who self-reported they had used 2-1-1 PrEP at least once were included. To verify age, individuals were asked to text message a photograph of an identification card with their date of birth. To verify PrEP use, individuals were asked to text message a photograph of their PrEP medication vial or pharmacy medication list to verify that they were prescribed PrEP. We did not specify a timeframe for the date of their prescription given individual differences in frequency of 2-1-1 PrEP use based on the frequency of sexual encounters. Interested individuals were invited to participate if they were able to describe the 2-1-1 dosing method. Use of 2-1-1 dosing was defined as having taken the full 2-1-1 dosing regimen at least one time. Participants qualified if they were formally prescribed 2-1-1 by a provider or if they had self-modified their daily PrEP to a 2-1-1 dosing strategy.

We developed a 35-item Qualtrics survey that was peer-reviewed by UCSF Center for AIDS Prevention Studies (CAPS) Methods Core (S1 File). Survey questions were developed specifically for this study. For the medication adherence questions, we modified a previously validated medication adherence question to pertain to the 2-1-1 PrEP context [20]. The survey was emailed to eligible participants starting in December 2019 with an estimation of approximately 30 minutes for completion.

The survey inquired about demographics and sex practices, PrEP use, facilitators of 2-1-1 PrEP use (including reasons for switching from once daily to 2-1-1; adherence to 2-1-1 dosing based on each component of the dosing strategy; strategies that participants have used to remember their 2-1-1 dosing regimen), PrEP preferences (choosing between PrEP attributes such as side effects, cost, and knowledge of provider), and barriers to 2-1-1 PrEP use (including unplanned sexual encounters, trouble remembering each dose of the 2-1-1 regimen, and lack of provider knowledge of 2-1-1 PrEP). On April 9, 2020, the survey was adapted and approved by the UCSF IRB to include SARS-CoV-2 related questions after the initiation of shelter-in-place orders these questions pertained to participants’ experience taking 2-1-1 PrEP during the pandemic (how shelter-in-place orders or policies in peoples’ area affected their dating and sex life; engagement in HIV prevention; ability to access care and resources pertaining to 2-1-1 PrEP). Participants received a US$30 electronic gift card upon survey completion. We calculated descriptive statistics to evaluate the study sample using means, percentages, and standard deviations (SDs).

## Results

### Demographics

Between December 2019 and June 2020, 140 individuals with a mean age of 38.2 years (SD = 11.6) participated, who were mainly male (95.0%); gay/queer/bisexual (85.7%); and non-Latino White (54.3%) (Table 1). About 72.1% reported that they had enough money to live comfortably and 90.0% reported any college education or higher. Geographically, 60.7% were located in the West or Northeast of the US [21]. Approximately 67.1% reported engaging in receptive anal sex and 63.6% reported insertive anal sex. A total of 34 (24.2%) participants completed the survey prior to start of shelter-in-place orders and 106 (75.7%) participants completed the survey after shelter-in-place.

**Table 1:** Summary of participant demographics.

		N = 140
Age, mean years (SD)		38.2 (11.6)
Gender, N (%)		
	Male	133 (95.0)
	Other	7 (5.0)
Race/Ethnicity, N (%)		
	Asian/Asian American (non-Latino)	20 (14.3)
	Black/African American (non-Latino)	7 (5.0)
	Latino	22 (15.7)
	White (non-Latino)	76 (54.3)
	Other (non-Latino)	15 (10.7)
Sexual Identity, N (%)		
	Gay or Queer	117 (83.6)
	Bisexual	3 (2.1)
	Other	20 (14.3)
Financial Security, N (%)		
	I have enough money to live comfortably	101 (72.1)
	I can barely get by on the money I have	37 (26.4)
	I cannot get by on the money I have	2 (1.4)
Education, N (%)		
	High school or less	13 (9.3)
	Any college	61 (43.6)
	Master’s degree or higher	65 (46.4)
	No response	1 (0.7)
US Region [21], N (%)		
	Midwest	9 (6.4)
	Northeast	21 (15.0)
	South	11 (7.9)
	West	64 (45.7)
	No response	35 (25.0)

### Facilitators to 2-1-1 PrEP use

From the 140 participants, 76.4% reported current use of 2-1-1 PrEP and 19.3% reported previous 2-1-1 PrEP use (Table 2). A total of 91.4% of respondents reported a history of daily PrEP use. Of those, 10.2% reported remembering a once-daily PrEP dose as difficult–very difficult and 13.3% rated their once-daily PrEP adherence as being fair–very poor. Participants had first heard about 2-1-1 PrEP from a healthcare provider (53.6%) and online (23.6%). The common reasons for switching from once-daily to 2-1-1 included having sex less frequently (63.6%), wanting to take fewer pills (46.4%), wanting to reduce medication costs (22.1%), and wanting fewer side effects (19.3%). Approximately 72.9% of participants felt confident that 2-1-1 was as effective as once-daily PrEP. Within the past three months, 87.9% reported using 2-1-1 PrEP at least once. Among those, 84.6% often–always remembered their double-dose, 91.9% and 90.2% often–always remembered their first and second single doses at 24 and 48 hours, respectively. Approximately 92.7% took their double-dose 2–24 hours before sex. The most common strategies to help participants remember their 2-1-1 dosing regimen included using a cell phone alarm/clock (37.9%), taking PrEP after a certain daily activity (28.6%), using a pillbox (20%), or using a mobile app (12.1%). Most participants (78.6%) reported feeling comfortable talking to a provider about PrEP, followed by 68.6% who felt comfortable talking to a PrEP navigator.

**Table 2:** PrEP use among participants.

Question	Response	N = 140
Are you currently using the 2-1-1 PrEP dosing method?, N (%)		
	Yes	107 (76.4)
	No	33 (23.6)
Why have you decided to switch from once-daily oral PrEP to on-demand oral PrEP?, N (%)		
	Because I am having sex less frequently	89 (63.6)
	Because I want to take fewer pills	65 (46.4)
	Because I want to reduce the cost of my pills	31 (22.1)
	Because I want fewer side effects	27 (19.3)
	Because my partner(s) changed	6 (4.3)
	Other	4 (2.9)
In the past 3 months, how many times have you taken on-demand dosing?, N (%)		
	0	16 (11.4)
	1–2	58 (41.4)
	3–4	39 (27.9)
	5 or greater	26 (18.6)
	No response	1 (0.7)
In the past 3 months, how frequently did you remember to take the double-dose 24 hours before your sexual encounter?^a^, N (%)		
	Never–Rarely	7 (5.7)
	Sometimes	12 (9.8)
	Often–Always	104 (84.6)
In the past 3 months, on average, how many hours before your sexual encounter did you take your double-dose?^a^, N (%)		
	0–1	9 (7.3)
	2–5	76 (61.8)
	6–10	16 (13.0)
	11–15	6 (4.9)
	16–20	5 (4.1)
	21–24	11 (8.9)
In the past 3 months how frequently did you remember to take your single dose 24 hours after the double-dose?^a^, N (%)		
	Never–Rarely	1 (0.8)
	Sometimes	7 (5.7)
	Often–Always	113 (91.9)
	No response	2 (1.6)
In the past 3 months how frequently did you remember to take your single dose 48 hours after the double-dose?^a^, N (%)		
	Never–Rarely	3 (2.4)
	Sometimes	8 (6.5)
	Often–Always	111 (90.2)
	No response	1 (0.8)
How confident do you feel about the effectiveness of on-demand PrEP versus once-daily PrEP?, N (%)		
	2-1-1 PrEP is less effective than once-daily PrEP	18 (12.9)
	2-1-1 PrEP is as effective as once-daily PrEP	102 (72.9)
	2-1-1 PrEP is more effective compared to once-daily PrEP	2 (1.4)
	No response	18 (12.9)
What are some strategies that have helped you remember your 2-1-1 doses?, N (%)		
	Use an alarm either on your cell phone or a clock	53 (37.9)
	Take medication after a certain daily activity (such as brushing your teeth or eating breakfast)	40 (28.6)
	Use pillbox (or medi-set)	28 (20.0)
	Use an application on your cell phone	17 (12.1)
	Writing a note to yourself	16 (11.4)
	Other	13 (9.3)
Which of the following healthcare team members would you feel comfortable talking to about 2-1-1 PrEP?, N (%)		
	Provider (Physician, Nurse Practitioner, Physician Assistant)	110 (78.6)
	PrEP Navigator	96 (68.6)
	Nurse	92 (65.7)
	Case Manager or Social Worker	64 (45.7)
	Pharmacist	63 (45.0)
	Peer Navigator	47 (33.6)
What barriers have you experienced with the 2-1-1 dosing strategy?, N (%)		
	Unplanned sexual encounters (i.e., missing the double dose 2–24 hours before a sexual encounter)	61 (43.6)
	Trouble remembering the dosing schedule after the double dose (i.e., at 24 and 48 hours after the double dose)	41 (29.3)
	Lack of healthcare provider knowledge about 2-1-1 dosing	33 (23.6)
	Cost associated with prescription and lack of insurance coverage	22 (15.7)
	Stigma	13 (9.3)
	Alcohol/drug use or mental health challenges	8 (5.7)
	Other	6 (4.3)
What challenges have you encountered related to your PrEP medication within the past month?^b^, N (%)		
	Lab tests	27 (25.5)
	Refills	20 (18.9)
	No appointments available	18 (17.0)
	Communication with your medical provider	12 (11.3)
	I haven’t experienced challenges	60 (56.6)
How has your city’s new Coronavirus policies affected how you seek out romantic or sexual partners?^b^, N (%)		
	Completely stopped sexual encounters	40 (37.7)
	Exclusively meeting with previous or known partners	23 (21.7)
	Having sex less frequently and with caution	18 (17.0)
	Unaffected by policies	12 (11.3)
	Sharing online content (messages, photos, and videos)	10 (9.4)
	No response	3 (2.8)

^a^N = 123.

^b^N = 106 (during shelter-in-place due to the SARS-CoV-2 pandemic).

### Preferred PrEP characteristics

When asked to choose between two different scenarios participants chose options that reflected three preferable general PrEP characteristics: **(1)** having few side effects- despite having higher dosing frequency (76.4%) versus lower dosing frequency (21.4%), despite being once-daily oral PrEP (67.1%) versus monthly injections (31.4%), or despite being more expensive (73.6%) versus less expensive (21.4%); **(2)** being less expensive- despite higher dosing frequency (65.0%) versus lower dosing frequency (32.9%), or despite being obtained with a prescription from a clinic (85.5%) versus buying over-the-counter at a pharmacy (15.0%); and **(3)** requiring fewer clinic visits (e.g., every three months) despite being once-daily oral (65.0%) versus monthly injections (34.3%). Approximately 79.3% of participants felt more comfortable seeing a provider who was knowledgeable about PrEP even if this person was different from their regular provider.

### Barriers to 2-1-1 PrEP use

Important 2-1-1 PrEP barriers included unplanned sexual encounters resulting in missing the double-dose 2–24 hours before sex (43.6%), trouble remembering dosing after the double-dose (29.3%), and lack of provider knowledge of 2-1-1 dosing (23.6%).

Of the 106 participants who completed the survey post shelter-in-place orders, 78.3% reported feeling somewhat to extremely concerned about the overall risk of SARS-CoV-2. Participants reported a mean of 1.9 (SD = 3.7) sex partners since their county had implemented SARS-CoV-2-related mandates. The top PrEP challenges during the pandemic included laboratory testing (25.5%), obtaining PrEP refills (either receipt of a refill authorization from a healthcare provider or processing of a refill from the pharmacy) (18.9%), being unable to get a healthcare provider appointment (17.0%), and not being able to communicate with their healthcare provider (11.3%). Finally, local city pandemic policies had resulted in participants completely stopping sexual encounters (37.7%), exclusively meeting prior partners (21.7%), or having sex less frequently but with caution (17.0%).

## Discussion

In this 2-1-1 PrEP survey, while the majority of participants reported previous daily PrEP use, reductions in sexual encounters, preference to take fewer pills, need to reduce cost, and desire to reduce side effects had resulted in the use of 2-1-1 PrEP. The overall preference for PrEP regimens with few side effects, low cost, and few clinic visits further underscores the importance for alternative PrEP regimens that are responsive to the needs and desires of patients. The study findings indicate high adherence to the 2-1-1 dosing strategy, with the vast majority of participants taking the double-dose within 2–24 hours of their sexual encounter. Facilitators of 2-1-1 PrEP adherence included phone alarms, pairing medication-taking with activities of daily living, or using a pillbox. Unplanned sexual encounters, trouble remembering the dosing schedule, and lack of healthcare provider knowledge of 2-1-1 PrEP accounted for the most frequently cited barriers to 2-1-1 PrEP use. In addition, other reported barriers to 2-1-1 PrEP use included cost, stigma, substance use, and mental health.

Our study and other US-based studies [9, 10] show a majority of participants had tried daily PrEP but subsequently switched to 2-1-1 PrEP. The data from our study similarly endorses findings from other studies [9, 10] that indicate that 2-1-1 PrEP dosing is a strategy used by people interested in reducing pill intake due to decreased frequency of sex. Similar to a study on early-adopters of 2-1-1 PrEP dosing, challenges were largely associated with adhering to the dosing schedule and planning sexual encounters [9]. In a prior study [9] and ours, 2-1-1 PrEP users reported an interest in a PrEP regimen that provided few side effects and the ability to reduce overall cost.

There are limited data on experiences of receiving PrEP services focused on 2-1-1 PrEP, especially during the SARS-CoV-2 pandemic. Participants noted a reduction in sex, which may explain switching to 2-1-1 PrEP use from daily dosing and reductions in monitoring during the SARS-CoV-2 pandemic that has been reported in recent studies [22–24]. Therefore, it is critical for providers, PrEP navigators, or clinic staff to educate MSM on 2-1-1 dosing. In addition to capturing facilitators and barriers to 2-1-1 PrEP, our data are important due to the description of sexual health and PrEP use during the SARS-CoV-2 pandemic, which placed additional demands on individuals engaging in HIV prevention. Without improving laboratory testing procedures (e.g., using standing laboratory orders or home-based testing [25, 26], easing PrEP refill authorizations and pharmacy processing procedures, enhancing communication with healthcare providers (e.g., via PrEP coordinators), and increasing ability to schedule visits with healthcare providers (e.g., using telehealth mechanisms), these barriers will continue to disrupt PrEP clinical services. Future studies should further explore PrEP access during the SARS-CoV-2 pandemic and how clinic limitations during this time influenced HIV prevention efforts.

Even though the majority of participants reported comfort in talking to a provider about PrEP, most preferred to discuss PrEP with a PrEP-knowledgeable provider and nearly a quarter of respondents reported the lack of provider knowledge as a barrier to 2-1-1 PrEP use. Additionally, similar to a recent national online study of MSM using non-daily PrEP [10], in our study a majority of participants came from the West and Northeast regions of the US [21], which may indicate the lack of 2-1-1 PrEP awareness, knowledge, or endorsement by providers in other regions. Therefore, we believe local public health departments and the CDC should endorse 2-1-1 PrEP dosing along with efforts to increase provider and patient awareness of 2-1-1 PrEP.

Some study limitations include a relatively small sample size of respondents who were mostly white, cisgender, MSM, who had attained higher education, were financially stable, and from the West and Northeast of the US [21]. Therefore, results may not be generalizable to other demographics and regions. Due to the cross-sectional nature of the study, we are unable to discern longitudinal changes in PrEP use. Finally, we relied on self-report, which is subject to recall and social desirability biases.

Results from IPERGAY and the Prévenir study have shown high efficacy of on-demand PrEP [8, 13]. Given the appeal of alternative dosing strategies, we urge healthcare providers, PrEP navigators, and other clinic staff to educate patients on 2-1-1 PrEP [7] and the option of switching between once daily and 2-1-1 dosing based on their preference and frequency of sexual encounters. During a pandemic, it is particularly important to provide alternatives to individuals wishing to engage in HIV prevention strategies given changes in frequency of sex.

## Supporting information

S1 File Quantitative survey. (PDF)
