# Supplementary material for: Facilitators and barriers of 2-1-1 HIV pre-exposure prophylaxis
Source: PLoS One. 2021 May 20;16(5):e0251917. doi: 10.1371/journal.pone.0251917 (PMC8136636; doi:10.1371/journal.pone.0251917)
Supplement: S1 File — (PDF) [file pone.0251917.s001.pdf]

Quantitative Survey for MSM and the use of 2-1-1 PrEP

Thank you for agreeing to participate in this study. As you know from our conversation when we were completing the consent form, the purpose of our study is to understand the facilitators and barriers of 2-1-1 PrEP (i.e., on-demand dosing or episodic dosing).

In the first section, we will ask questions about demographics.

1) How old are you?
2) What city and state do you currently reside in?
3) Select one or more of the following **racial categories** that apply to you (check all that apply)
    a. American Indian / Alaskan Native
    b. Native Hawaiian / Pacific Islander
    c. Black / African American
    d. Asian / Asian American
    e. White
    f. Multiracial / Multicultural
    g. Decline to answer
4) How would you describe your **ethnic** background?
    a. Latino
    b. Non-Latino
    c. Decline to answer
5) What **sex** were you assigned at birth?
    a. Male
    b. Female
    c. Intersex
    d. Decline to answer
6) What is your **current gender identity**? (Check all that apply.)
    a. Male
    b. Female
    c. Transgender Male
    d. Transgender Female
    e. Genderqueer
    f. Additional category (specify): ________________________________
    g. Decline to answer
7) What is your **sexual orientation**?
    a. Heterosexual or straight
    b. Gay
    c. Lesbian
    d. Bisexual
    e. Queer

f. Additional category (specify): ________________________________
g. Decline to answer

8) Which of the following statements best describes your **financial situation**?
    a. I have enough money to live comfortably
    b. I can barely get by on the money I have
    c. I cannot get by on the money I have
    d. Decline to answer
9) What is the highest level of **education** you have completed?
    a. No formal education
    b. Did not graduate from high school
    c. High school graduate or GED
    d. 2 years of college/AA degree/technical school training
    e. College graduate (BA/BS)
    f. Master's degree
    g. Doctorate/Medical degree/Law degree
    h. Decline to answer

In the next section, we are going to ask you about once-daily oral pre-exposure prophylaxis (PrEP) versus 2-1-1 PrEP dosing.

10) Have you ever used once-daily PrEP?
    a. Yes
    b. No
    c. Unsure
    d. Decline to answer
11) *(If "yes" to Q10)* If you are were on once-daily PrEP and switched to 2-1-1 PrEP, why did you decide to change?
    a. Because I want fewer side effects
    b. Because I want to take fewer pills
    c. Because I am having sex less frequently
    d. Because I want to reduce the cost of my pills
    e. Because my partner(s) changed
    f. Other: ________________________________
12) Are you currently using the 2-1-1 PrEP dosing method?
    a. Yes
    b. No
    c. Decline to answer
13) Have you previously used the 2-1-1 PrEP dosing method?
    a. Yes
    b. No
    c. Unsure
    d. Decline to answer

14) (*If answer "yes" to Q12 & Q13*), In the past 3 months how many times have you taken 2-1-1 dosing?
   a. 0
   b. 1-2
   c. 3-4
   d. 4-5
   e. 6 or greater
   f. Decline to answer
15) (*If >0 on Q14*) In the past 3 months how frequently did you remember to take the double dose 24 hours before your sexual encounter?
   a. Never
   b. Rarely
   c. Sometimes
   d. Often
   e. Always
   f. Decline to answer
16) (*If >0 on Q14*) In the past 3 months, on average, how many hours before your sexual encounter did you take your double dose?
   a. 0-1
   b. 2-5
   c. 6-10
   d. 11-15
   e. 16-20
   f. 21-24
   g. Decline to answer
17) (*If >0 on Q14*) In the past 3 months how frequently did you remember to take your single dose 24 hours after the double dose?
   a. Never
   b. Rarely
   c. Sometimes
   d. Often
   e. Always
   f. Decline to answer
18) (*If >0 on Q14*) In the past 3 months how frequently did you remember to take your single dose 48 hours after the double dose?
   a. Never
   b. Rarely
   c. Sometimes
   d. Often
   e. Always
   f. Decline to answer
19) How did you first hear about 2-1-1 PrEP? Check all that apply.

a. Clinician or healthcare provider
b. Friends or Family
a. News (Online or paper news sources)
b. Online media (apps, podcasts, social networking, websites, etc)
c. Online Marketing (ads, pop-ups and banners online)
d. Paper advertisement or billboards
e. Other
f. Please specify: _________________________________
g. Decline to answer

20) How confident do you feel about the effectiveness of 2-1-1 PrEP versus once-daily PrEP? a. 2-1-1 PrEP is less effective than once-daily PrEP
b. 2-1-1 PrEP is as effective as once-daily PrEP
c. 2-1-1 PrEP is more effective compared to once-daily PrEP
d. Decline to answer

21) What are some strategies that have helped you remember your 2-1-1 doses? Select all that apply.
a. Use pillbox (or medi-set)
b. Take medication after a certain daily activity (such as brushing your teeth or eating breakfast)
c. Use an alarm either on your cell phone or a clock
d. Use an application ("app") on your cell phone
e. Ask your family or friend to remind you
f. Writing a note to yourself
g. Other: _____________________________________
h. Decline to answer

22) Which of the following healthcare team members would you feel comfortable talking to about PrEP? Select all that apply.
a. Provider (Physician, Nurse Practitioner, Physician Assistant)
b. Nurse
c. Pharmacist
d. PrEP Navigator
e. Case Manager
f. Social Worker
g. Peer Navigator
h. Other: _________________________________
i. Decline to answer

23) What barriers have you experienced with the 2-1-1 dosing strategy? Select all that apply. a. Lack of insurance coverage
b. Lack of healthcare provider knowledge about 2-1-1 dosing
c. Cost associated with prescription
d. Unplanned sexual encounters (i.e., missing the double dose 2-24 hours before a

sexual encounter)

e. Trouble remembering the dosing schedule after the double dose (i.e., at 24 and 48 hours after the double dose)
f. Alcohol or substance use
g. Mental health challenges
h. Stigma
i. Other: ________________________________
j. Decline to answer

In the next section, we will ask you to choose between two options in various scenarios. Please let us know what your preference is.

24) Would you prefer to take PrEP with **lower dosing frequency** that may have **more side effects**, or would you prefer to take PrEP that may have **less side effects** but has a **higher dosing frequency**?
    a. Prefer PrEP with lower dosing frequency that may have more side effects
    b. Prefer PrEP with higher dosing frequency that may have less side effects
    c. Decline to answer
25) Would you prefer to take PrEP with a **lower dosing frequency** that may be **more expensive**, or would you prefer to take PrEP with a **higher dosing frequency** that may be less **expensive**?
    a. Prefer to take PrEP with a lower dosing frequency that may be more expensive
    b. Prefer to take PrEP with a higher dosing frequency that may be less expensive
    c. Decline to answer
26) Would you prefer to take **oral once-daily** PrEP that may have **fewer side effects** or would prefer to take **a monthly PrEP injection** that may have **more side effects**?
    a. Prefer to take oral once-daily PrEP with the possibility of fewer side effects
    b. Prefer to receive a monthly injection with the possibility of more side effects
    c. Decline to answer
27) Would you prefer a more expensive PrEP regimen that may have **fewer side effects,** or would you prefer a less expensive PrEP regimen that may have **more side effects**?
    a. Prefer more expensive PrEP dosing regimen that may have fewer side effects
    b. Prefer a less expensive PrEP regimen that may have more side effects
    c. Decline to answer
28) Would you prefer to buy PrEP **over the counter** at a pharmacy that may be **more expensive,** or would you prefer obtaining a prescription for PrEP **during clinic hours** that may be **less expensive**?
    a. Prefer to buy over the counter at a pharmacy that may be more expensive
    b. Prefer to obtain a prescription in a clinic that may be less expensive
    c. Decline to answer
29) Would you prefer to visit the clinic **once a month** for a **PrEP injection** or would you prefer to visit the clinic **once every three months** for **oral PrEP**?

a. Prefer to visit the clinic once a month for an injection
b. Prefer to visit clinic once every three months and take oral PrEP
c. Decline to answer

30) Would you feel comfortable getting your PrEP prescription from your **regular primary care provider**, who may be **less knowledgeable** about PrEP, or would you feel more comfortable seeing **a different provider** for PrEP who is **more knowledgeable** about PrEP?
a. Feel more comfortable with a regular primary care provider who may not be knowledgeable about PrEP
b. Feel more comfortable with a different provider who is more knowledgeable about PrEP
c. Decline to answer

In the next section, we will ask you questions about the novel Coronavirus, the virus responsible for COVID-19, and how the global pandemic has affected your sexual health.

31) How concerned are you about contracting Coronavirus?
a. Not at all concerned
b. Slightly concerned
c. Somewhat concerned
d. Moderately concerned
e. Extremely concerned
f. Decline to answer

32) What challenges have you encountered related to your PrEP medication within the past month?
a. Refills
b. Lab tests
c. Communication with provider
d. No appointments available
e. I haven’t experienced challenges
f. Other

33) Since the initiation of your city’s new Coronavirus policies (i.e., shelter in place, self-quarantine, etc.) how difficult is it to get an HIV test?
a. Not at all difficult
b. Slightly difficult
c. Moderately difficult
d. Slightly difficult
e. Extremely difficult
f. I have not attempted to get an HIV test

g. Decline to answer

34) Since the initiation of your city’s new Coronavirus policies, how many romantic or sexual partners have you had?
_____ # (text box)

35) How has your city’s new Coronavirus policies affected how you seek out romantic or sexual partners?
a. Completely stopped sexual encounters
b. Exclusively meeting with previous or known partners
c. Having sex less frequently and with caution
d. Sharing online content (messages, photos, and videos)
e. Unaffected by policies
f. No response
